# Assessment of differential intraocular pressure response to dexamethasone treatment in perfusion cultured Indian cadaveric eyes

**DOI:** 10.1038/s41598-020-80112-8

**Published:** 2021-01-12

**Authors:** Ravinarayanan Haribalaganesh, Chidambaranathan Gowri Priya, Rajendrababu Sharmila, Subbaiah Krishnadas, Veerappan Muthukkaruppan, Colin E. Willoughby, Srinivasan Senthilkumari

**Affiliations:** 1 Department of Ocular Pharmacology, Aravind Medical Research Foundation, #1, Anna Nagar, Madurai, Tamilnadu 625020 India; 2 Department of Immunology and Stem Cell Biology, Aravind Medical Research Foundation, #1, Anna Nagar, Madurai, 625020 India; 3 Glaucoma Clinic, Aravind Eye Hospital, #1, Anna Nagar, Madurai, 625020 India; 4 Genomic Medicine Group, Biomedical Sciences Research Institute, Ulster University, Coleraine, Northern Ireland UK

**Keywords:** Molecular biology, Medical research, Pathogenesis

## Abstract

The purpose of the present study was to assess the differential intraocular pressure response (IOP) to dexamethasone (DEX) treatment at two dose levels (100 or 500 nM) in perfusion cultured Indian cadaveric eyes to investigate glucocorticoid (GC) responsiveness. In a human organ-cultured anterior segment (HOCAS) set-up, the eye pressure was monitored for every 24 h post DEX infusion (100 or 500 nM) or 0.1% ethanol treatment for 7 days after baseline stabilization. The expression of DEX-inducible proteins such as myocilin and fibronectin in HOCAS-TM tissues was assessed by immunostaining. Elevated IOP was observed in 6/16 eyes [Mean ± SEM (mΔIOP): 15.50 ± 1.96 mmHg; 37.5% responders] and 3/15 eyes (Mean ± SEM mΔIOP: 10 ± 0.84 mmHg; 20% responders) in 100 nM and 500 nM dose groups respectively. Elevated IOP in GC responder eyes was substantiated with a significant increase in myocilin (11.8-fold; *p* = 0.0002) and fibronectin (eightfold; *p* = 0.04) expression as compared to vehicle-treated eyes by immunofluorescence analysis. This is the first study reporting the GC responsiveness in Indian cadaveric eyes. The observed GC response rate was comparable with the previous studies and hence, this model will enable us to investigate the relationship between differential gene expression and individual GC responsiveness in our population.

## Introduction

Glucocorticoids (GC) have been the mainstay for the management of inflammatory eye diseases due to its potent anti-inflammatory, anti-angiogenic and immune-modulatory properties^1^. Chronic use of GC induces ocular hypertension (GC-OHT) and GC-induced glaucoma (GIG) in susceptible individuals (GC responders)^2,3^. It is reported that 40% of the patients in the general population showed an increased intraocular pressure (IOP) with topical dexamethasone use, of which, 6% are likely to develop glaucoma^2,4,5^. More than 90% of the glaucoma patients are GC responders which further complicates clinical management as a GC response can effect IOP control increasing the susceptibility of losing vision in these patients^3,6^. Both GIG and primary open angle glaucoma (POAG) share similarities in clinical presentation such as an open angle, increased IOP, characteristic optic neuropathy and loss of peripheral vision^7,8^. However, the molecular mechanisms for the pathogenesis of GIG are not completely understood^3^.

The trabecular meshwork (TM) is an important component in the conventional aqueous humor outflow pathway which plays a crucial role in maintaining the IOP homeostasis. GCs are known to induce alterations in TM structure and function including inhibition of cell proliferation and migration^9^, cytoskeletal rearrangement (formation of cross-linked actins (CLANs)^10–12^, increased TM cell and nuclear size^7^, accumulation of excessive extracellular matrix^11,13^, decreased phagocytosis^14^ and alterations in cellular junctional complexes^15^. These cellular, biochemical and morphological changes result in increased outflow resistance and decreased outflow facility.

Several in vitro*, *in vivo and ex vivo models have been developed to understand the pathogenesis of GI-OHT/GIG at a cellular and molecular level^16^. Perfused organ-cultured anterior segment (OCAS) has been used as a standard ex vivo model to examine the aqueous outflow pathway in glaucoma research for nearly 30 years^17–20^. This model serves as an intermediate between in vitro and in vivo systems and offers unique opportunity to study the physiology, biochemistry and morphology of the outflow pathway for a number of days (up to 1 month) in viable tissues^19^. In addition, the GC responder rate of perfusion cultured non-glaucomatous human eyes was 30% which is very close to the response rate observed in human subjects^2,10^. However, such a high GC responsiveness rate was not reported by other groups^18^.

To our knowledge, no studies have been reported to date in the Indian population. Therefore, the purpose of the present study was to utilize the human organ-cultured anterior segment (HOCAS) ex vivo model to investigate the GC responsiveness of Indian cadaveric eyes to dexamethasone (DEX) treatment at two dose levels (100 or 500 nM). This study identified 37.5% of eyes showed a GC response with the 100 nM dose of DEX and interestingly, a 500 nM DEX dose resulted in a 20% response rate. No dose-dependent increase in the GC response rate was observed in the studied eyes despite of increasing the DEX dose to fivefold. The elevated IOP of the GC responder eyes was substantiated with a significant increase in mean fluorescence intensity of myocilin (11.8-fold; *p* = 0.0002) and fibronectin expression (eightfold; *p* = 0.04) as compared to vehicle-treated and non-responder eyes by immunofluorescence analysis. Thus, the HOCAS model provides a platform to investigate the molecular mechanisms contributing to differential responses in the TM to GCs and the heterogeneity of glucocorticoid receptor (GR) signaling both in health and diseased conditions.

## Results

A total of 43 human donor eyes (7 paired; 29 single eyes) with the mean age of 73.0 ± 9.50 years were used for the present study and their demographic details are summarized in Supplementary Table S1. All anterior segments were cultured within 48 h of death (mean ± SD: 29.71 ± 14.89 h).

### Differential IOP response to DEX treatment

Human eyes were perfusion cultured with either DEX or 0.1% ethanol (ETH) as vehicle control for 7 days as described previously^10,21^. The DEX-induced elevated IOP was studied at two dose levels (100 nM and 500 nM) to check the dose-dependent response rate (RR). Out of 43 eyes, 16 eyes received 100 nM DEX, 15 eyes received 500 nM DEX and 12 eyes received 0.1% ethanol (ETH) for 7 days. Elevated IOP was observed in (6/16; RR = 37.5%) eyes in 100 nM dose group whereas in 500 nM dose group, 3/15 eyes showed a significant elevated IOP (RR = 20%).

**Figure 1:**
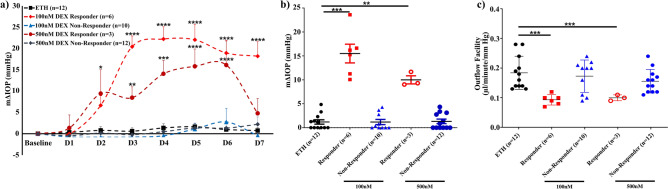
Effect of DEX on IOP. (**a**) The mean ± SEM of ∆IOP of ETH (vehicle control), DEX-treated responder and non-responder eyes were plotted over time. The eye pressure of the anterior segments in culture was acquired using Power Lab data acquisition system (AD Instruments, NSW, Australia) and analyzed using LabChart Pro software (ver.8.1) as described in detail in methods section. The basal IOP on day 0 (before DEX treatment) was set at 0 mmHg. In 100 nM dose group, treatment with DEX showed a significant elevated IOP in 6/16 eyes (Mean ± SEM—mΔIOP: 15.50 ± 1.96 mmHg; Response rate: 37.5%) whereas in 500 nM dose group, 3/15 eyes showed a very significant elevated IOP (Mean ± SEM—mΔIOP: 10 ± 0.84 mmHg; Response rate: 20%). ETH treated eyes showed mean ± SEM—mΔIOP of 0.92 ± 0.54 mmHg. Data were analyzed by unpaired 2-tailed Student’s t test on each treatment day. *p < 0.05; **p < 0.001; ***p < 0.0001; ****p < 0.00001. Frequency Plot of the (**b**) IOP data and (**c**) Outflow facility data. The m∆IOP and outflow facility of ETH treated, DEX-responder and DEX-non-responder groups were plotted for both 100 and 500 nM dose groups. The m∆IOP was increased after DEX treatment in responder eyes as compared to non-responder and ETH-treated eyes whereas outflow facility was decreased significantly after DEX treatment (100 and 500 nM) in responder group as compared to non-responder and ETH-treated groups.

A significant and progressive increase in IOP was observed in DEX-responder eyes after treatment with the mean Δ (± SEM) IOP of 15.5 ± 1.96 mmHg in 100 nM dose group and 10.0 ± 0.84 mmHg in 500 nM dose group. The mean ΔIOP in DEX-non-responder eyes was found to be 1.19 ± 0.54 mmHg and 1.32 ± 0.47 mmHg in 100 nM and 500 nM DEX respectively. The vehicle treated eyes remained stable throughout the study and the mean pressure was well below 5 mmHg (1.19 ± 0.46 mmHg). The DEX-treated responder eyes were statistically different from non-responder eyes and vehicle-treated eyes (*p* < 0.001) (Fig. 1a,b,c). In addition, the elevated IOP between 100 and 500 nM DEX-treated responder eyes were statistically significant (*p* = 0.04).

The mean (± SEM) basal outflow facility of total eyes (n = 43) was found to be 0.17 ± 0.01 µl/minute/mmHg at the perfusion rate of 2.5 µl/minute. In the DEX 100 nM dose group, the basal outflow facility of GC responder and GC non-responder eyes were 0.19 ± 0.04 and 0.16 ± 0.02 µl/minute/mmHg respectively; whereas in 500 nM dose group, the basal outflow facility of GC responder and GC non-responder eyes were 0.16 ± 0.01 and 0.16 ± 0.01 µl/min/mmHg respectively. The basal outflow facility of ETH treated eyes was calculated to be 0.19 ± 0.01 µl/min/mmHg. There was no significant difference found among three groups which further confirms that the observed elevated IOP in GC responder eyes was purely due to DEX treatment and not due to endogenous differences in the outflow facility and IOP of the studied eyes. The raw data of IOP and outflow facility of all the studied eyes are summarized in Supplementary Table S2A, 2B respectively.

### Effect of DEX on morphology and tissue viability

**Figure 2:**
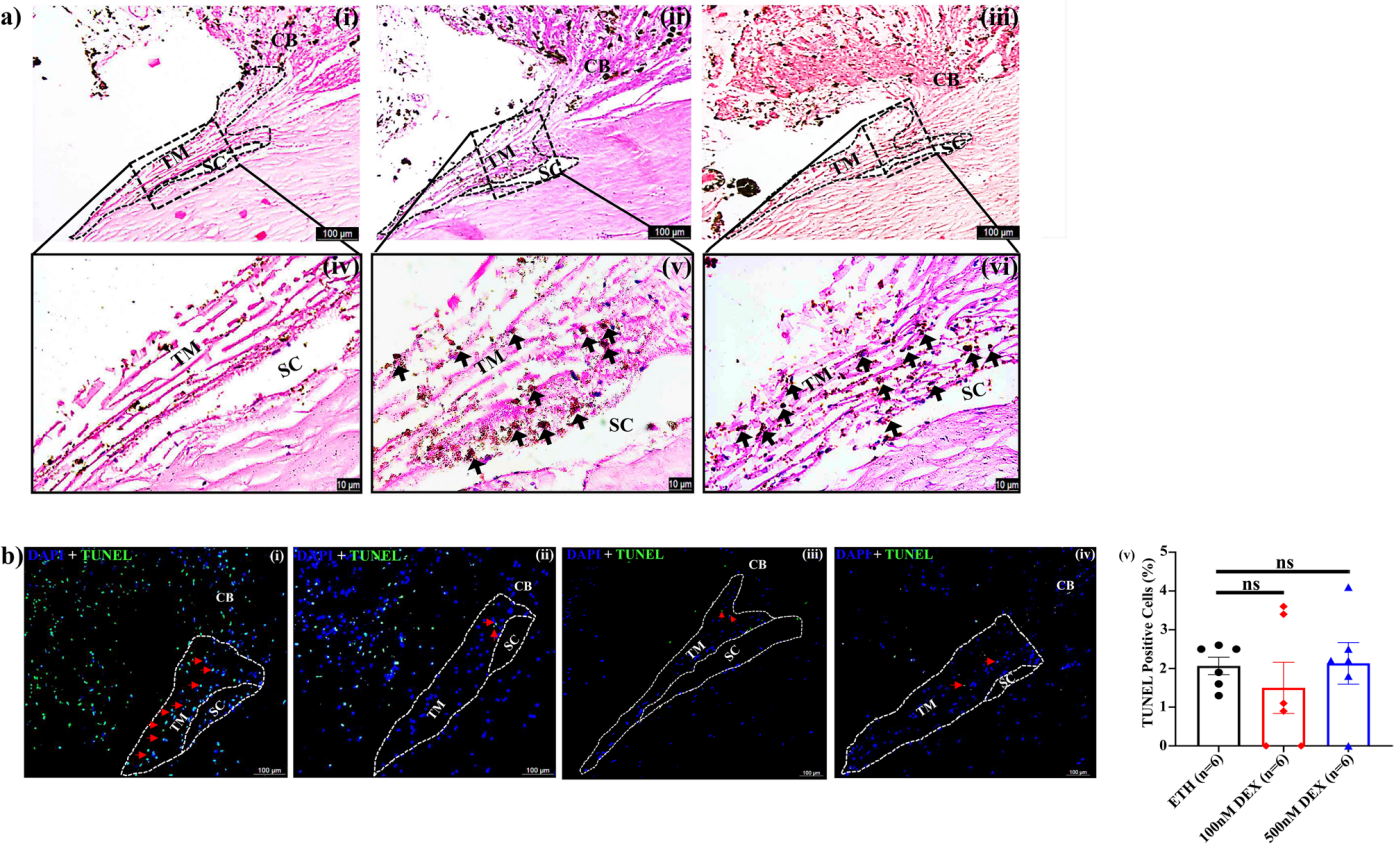
Effect of DEX on Morphology and Tissue viability. (**a**) Histology of Anterior Segment received (i, iv) 0.1% ETH, (ii, v) 100 nM DEX and (iii, vi) 500 nM DEX treatment for 7 days. High deposition of extracellular debris was found in DEX-treated eyes (indicated in black color arrow) (v, vi) as compared to vehicle-treated eyes (iv). (**b**) Representative images showing TUNEL positivity in (i) positive control (DNAase I treated), (ii) 0.1% ETH treated eyes, (iii) 100 nM DEX (iv) 500 nM DEX treated eyes and (v) Graph showing the percentage of TUNEL positive cells in ETH (n = 6), 100 nM DEX (n = 6) and 500 nM DEX-treated eyes (n = 6). No significant toxicity was observed after DEX treatment. The TUNEL positivity is indicated in red color arrow. TM-Trabecular meshwork; SC-Schlemm’s canal and CB- Ciliary body.

A deposition of high extracellular debris was found in DEX-treated anterior segments as compared to vehicle-treated eyes (Fig. 2a). TUNEL assay revealed that the tissue viability was not affected by either DEX/ETH treatment (Fig. 2b).

### DEX-induced myocilin and fibronectin expression in HOCAS-TM tissues

**Figure 3:**
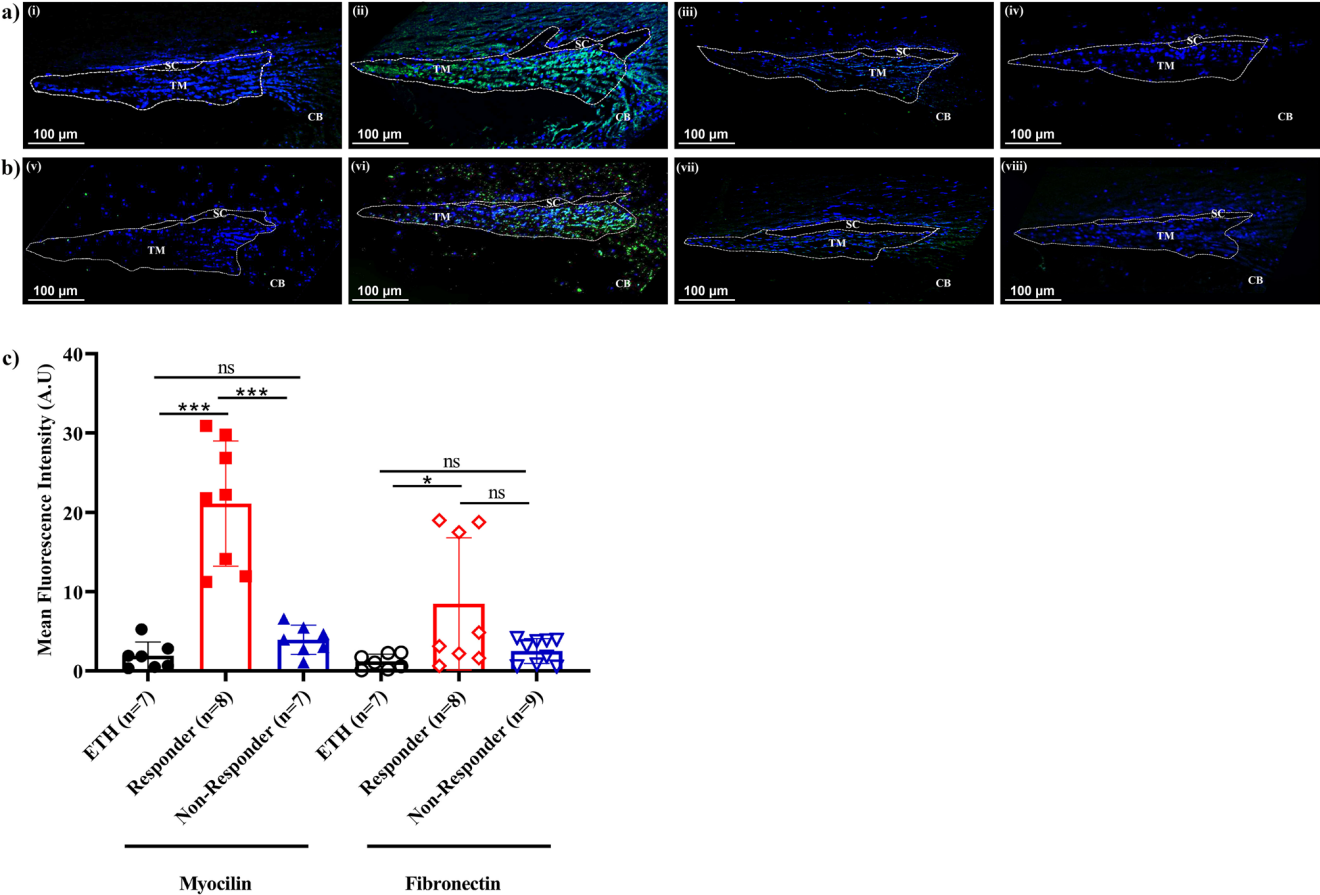
Effect of DEX on Myocilin and Fibronectin Expression in HOCAS-TM Tissues. Representative confocal images of (**a**) myocilin and (**b**) fibronectin expression (green) in ETH (Vehicle)-treated (i,v), DEX-responder (ii, vi), non-responder (iii, vii) eyes and negative control (no primary antibody) (iv, viii), DAPI (Blue): Nuclear counterstain. (**c**) Quantification of mean fluorescence intensity of myocilin and fibronectin expression in HOCAS-TM tissues of ETH- treated (n = 7), DEX-responder (n = 7) and non-responder eyes (7–9). Fluorescence images of five consecutive sections of TM from DEX treated and vehicle control eyes were analyzed and quantified for myocilin and fibronectin expression (green) in TM using Image J software [https://imagej.nih.gov/ij/]. A significant increase in mean fluorescence intensity of myocilin (*p* = 0.0002) and fibronectin (*p* = 0.04) was found in GC responder eyes as compared to vehicle-treated eyes. Data are shown as mean ± SEM. *p < 0.05; ****p < 0.00001; Un-paired *t* test. TM-Trabecular meshwork; SC-Schlemm’s canal and CB-Ciliary body.

The effect of DEX on the expression of myocilin and fibronectin was investigated in TM tissues after HOCAS experiment by immunofluorescence analysis and the representative images are shown in Fig. 3a,b. Interestingly, upon quantification a significant increase in mean fluorescence intensity of myocilin expression was found in GC responder eyes (11.8-fold) as compared to vehicle-treated eyes (*p* = 0.0002) and GC non-responder eyes (*p* = 0.0004) whereas fibronectin showed a eightfold increase in its expression (*p* = 0.04) (Fig. 3c). This clearly indicates that the elevated IOP correlates with a significant increase in myocilin and fibronectin expression.

## Discussion

The present study demonstrated the differential IOP response of perfused human cadaveric eyes to DEX treatment at two dose levels (100 and 500 nM). The dose of 100 nM DEX was chosen based on the concentration of DEX found in the aqueous humor of the human eyes following topical administration of a single drop of 0.1% DEX formulations^22^. The fivefold increase in DEX was chosen to explore any dose-dependent variations in IOP response and also in some in vitro studies, 500 nM dose was used to explore the gene /proteomic alterations in response to DEX treatment in cultured human TM cells^23–26^.

The change in IOP in response to DEX treatment (“ΔIOP”, defined as the maximum IOP after treatment minus the baseline IOP) was determined as a positive response for all studied eyes. The “GC responder eyes” were defined according to the criteria described earlier with a positive ΔIOP ≥ 5 mmHg above baseline IOP following DEX treatment and the “GC- non-responder eyes” are those with their ΔIOP ≤ 5 mmHg above baseline IOP^10^. Based on these criteria, the GC responsive eyes of the present study were found to be 37.5% at 100 nM dose. Most of the eyes were found to be moderate responders (ΔIOP range: 8–15 mmHg). The observed GC response rate for 100 nM dose is comparable with the previous observation in perfusion cultured human eyes^10^.

GC responsiveness mainly depends upon the potency, route of administration, dosage and duration of GC exposure^3^. Interestingly, in our study by increasing the dose of DEX by fivefold (from 100 to 500 nM) did not show any dose-dependent increase in GC-response rate for the 500 nM group (20%) and their response rate was 1.9-fold less as compared to 100 nM dose group (37.5%). The inability of higher dose to elicit a dose-dependent increase in 500 nM dose group could be due to the saturation effect of GC receptors which may be responsible for the observed progressive decline in IOP response in the present study (Fig. 1).

In this study, a 7 days treatment regimen was chosen to investigate the IOP response of the studied eyes to DEX because the observed lag time of high IOP was between 3 and 5 days. Therefore, a 7 days treatment regimen was sufficient to get a positive GC response in our studied eyes. In contrast, the previous study observed the lag time of high IOP after 5–6 days and hence the eyes needed longer exposure time of 10–15 days for DEX treatment^10^. This could be due to variations in the flow rate used, as the present study utilized the flow rate of 2.5 µl/min whereas in the Clark et al.^10^, study, 2 µl/minute was used. The flow rate between 2 and 5 µl/min is mainly used to mimic the physiological human aqueous humor turnover rate and it is also well documented that this flow rate range preserves the health of the TM in perfusion culture^17^. The tissue viability data of the present study also supports this finding.

DEX-treatment is known to induce the expression of several genes and proteins in the TM including myocilin^3^. Myocilin is a glycoprotein and its physiological function is not clearly understood in the TM and in other ocular tissues^27^. It was first identified as a major GC-responsive gene and protein in the TM and is also found in the aqueous humor of patients with POAG^27,28^. Therefore, in the present study, the expression of myocilin in the HOCAS-TM tissues and its association with elevated IOP was investigated. Interestingly, our data revealed that a highly significant increase in myocilin expression (11.8-fold) was observed in TM region of the responder eyes compared to vehicle-treated eyes (*p* = 0.0002); and there was a fourfold increase in myocilin expression in GC responder eyes (*p* = 0.0004) compared with GC non-responder eyes. Such induction of myocilin upon DEX treatment was reported previously in trabecular meshwork monolayer cells and cultures of anterior segments, and showed the increase in myocilin expression was time and dose-dependent and correlated with the timing and increase of IOP^29,30^. It is well accepted fact that, the induction of myocilin in response to DEX treatment may not contribute to IOP raise in GC-OHT^31^. Recently, it is demonstrated that the induction of myocilin is mediated through a secondary activation of an inflammatory signaling pathway involving calcineurin and transcription factor NFATC1^25^.

Glucocorticoids are known to induce ECM changes in TM which are responsible for the aqueous outflow resistance in POAG. One such ECM protein is fibronectin which gets accumulated in the TM upon DEX treatment^29^. In the present study, we also found a eightfold increase in mean fluorescence intensity of fibronectin expression in TM of the GC-responder eyes as compared to vehicle-treated eyes (*p* = 0.04; n = 7) by immunohistochemical analysis. This corresponds to a previous study in perfusion cultured human eyes wherein a denser distribution of fibronectin was seen after DEX treatment in the JCT/inner endothelial cells of TM tissues of GC responder eyes as compared to control eyes^10^. Very recently, an increased fibronectin and other ECM proteins were also found in the TM region of *ex-vivo* cultured human corneo-scleral segments after DEX treatment^32^. This observation clearly supports the fact that GC responsiveness may be associated with fibronectin induction and ECM alterations in the TM.

It is interesting to note that, to date, 20 isoforms of fibronectin are generated in humans due to alternative splicing and it is not well understood how these fibronectin isoforms contributes to elevate IOP in glaucoma including GIG^33^. The induction of fibronectin in response to GCs could be mediated through TGF-β2 and there are elevated levels of TGF-β2 in cultured TM cells exposed to GC treatment and in a mouse model of GC-induced glaucoma^30^. Recent study suggested that the expression of constitutively active fibronectin-extra domain A itself is capable of inducing elevated IOP through TGF-β signaling in mice^34^. In the present study, the levels of TGF-β2 were not measured in perfusate of HOCAS (either by ELISA or western blotting), however, it would be worth investigating the relationship between TGF-β2 mediated fibronectin inductions upon DEX treatment and IOP response in perfusion cultured human anterior segments.

The limitations of the present study include that only limited number of paired eyes were available to assess the differential IOP response to DEX treatment due to high experimental rejection rate. The high experimental rejection rate was due to unstable baseline pressure during stabilization period. Therefore, both single eyes and paired eyes with stable baseline pressure were used for the present study. All the eyes used in the present study were from elderly donors (mean age of 73.0 ± 9.50 years) with no known ocular history. Hence, the prior history of GC treatment for any inflammatory conditions for the studied donors was not known. In addition, the observed GC sensitivity might have been greatly influenced with aged donors used in the present study.

In conclusion, this is the first study demonstrating the GC response rate in perfused human cadaveric eyes of Indian origin. The observed GC response rate at a 100 nM dose of DEX was similar to previously reported studies in perfusion cultured human eyes and clinical subjects. Increasing the DEX dose by fivefold showed no dose-dependent increase in GC-response rate. The known DEX-inducible proteins, such as myocilin and fibronectin, were found to have a positive association with elevated IOP and GC responsiveness. Thus, this study raises the possibility of identifying genes and proteins which are uniquely expressed by the GC responder eyes in the Indian population and further understanding into how these genes contribute to the differential responsiveness to GC therapy in all populations.

## Materials and methods

### Ethical statement

The donor eyes not suitable for corneal transplantation due to insufficient corneal endothelial cell count were included in this study. The written informed consent of the deceased donor or next of kin was also obtained. The study protocol was approved by the Institutional Ethics Committee of Aravind Medical Research Foundation (ID NO. RES2017006BAS) and was conducted in accordance with the tenets of the Declaration of Helsinki.

### Human donor eyes

Post-mortem human eyes were obtained from the Rotary Aravind International Eye Bank, Aravind Eye Hospital, Madurai, India. The donor eyes were enucleated within 4 h of death (mean elapsed time between death and enucleation was 2.86 ± 1.18 h) and kept at 4 °C in the moist chamber until culture. All eyes were examined under the dissecting microscope for any gross ocular pathological changes and only eyes without such changes were used for the experiments. The presence or absence of glaucomatous changes in the study eyes were confirmed by histo-pathological analysis of the posterior segments as described earlier by our group (data not shown)^35^. The characteristics of donor eyes for this study is summarized in Supplementary Table S1.

### DEX–induced ocular hypertension (DEX-OHT) in perfused human cadaveric eyes

Paired / single post-mortem eyes were used to establish HOCAS by the method as described earlier^36^. Briefly, human donor eyes were dissected out after removing vitreous, lens and iris leaving the ciliary body from the donor eyes. Then the anterior segments were placed in a specially designed Petri dish with the cornea side up. The segments were secured with plastic O ring and perfused continuously with Dulbecco’s Modified Eagle’s Medium (DMEM, containing 4500 mg glucose/L, l-glutamine, NaHCO_3_ and pyridoxine HCl, Sigma-Aldrich, St. Louis, MO) supplemented with gentamycin (15 mg/L, Sigma-Aldrich, St. Louis, MO) and antibiotic/antimycotic solution (penicillin G, 100 U/ml; streptomycin sulphate, 100 μg/ml; amphotericin B, 0.25 μg/ml: Sigma-Aldrich, St. Louis, MO) at a flow rate of 2.5 μl/minute using the Harvard Apparatus slow infusion pump (PHD2000, MA,USA). All eyes were allowed to stabilize in the HOCAS set up for at least 24–72 h. The donor eyes which failed to stabilize within 72 h of perfusion culture and with their outflow facility outside the range of 0.1–0.3 µl/minute/mmHg were excluded from the study.

After baseline stabilization, one eye of each pair or designated single eyes received 5 ml of either 100 or 500 nM DEX at the flow rate of 200 µl/minute and the contralateral eye of the paired eyes or designated single eyes received 0.1% ethanol (ETH) as vehicle control and resumed the flow rate to 2.5 µl/minute with respective treatments until 7 days (3 doses). The eye pressure was monitored continuously using pressure transducers (APT 300 Pressure Transducers, Harvard Apparatus, MA, USA) with data recorder (Power Lab system (AD Instruments, NSW, Australia) with LabChart Pro software (ver.8.1).

### Measurement of pressure change after DEX treatment

The intraocular pressure (IOP) was calculated every hour as the average of 6 values recorded every 10 min, beginning 4 h before the drug infusion and continued for the duration of the culture. The average IOP of 4 h before drug infusion was taken as baseline IOP for calculation. Mean IOP was calculated for every day (24 h) after respective treatments. Then ΔIOP was calculated using the formula: (Actual IOP averaged over 24 h—Basal IOP of individual eyes before drug treatment). The increase in IOP in response to DEX treatment was examined for all treated eyes. The eyes were categorized as GC responder (mean ΔIOP was > 5 mmHg from the baseline) and non-responder eyes (mean ΔIOP ≤ 5 mmHg from the baseline) after DEX treatment for 7 days as described earlier^37^. The raw data of the IOP and the outflow facility are given in Supplementary Table S2A and 2B respectively.

### Morphological analysis of the outflow tissue

At the end of the drug treatment, anterior segments were fixed by perfusion with 4% paraformaldehyde. Perfusion fixed anterior segments were processed for histological examination using standard protocol. TM was considered normal if trabecular cells remained in their usual position on the lamellae (subjective assessment), and no or minor disruption of the juxtacanalicular tissue and trabecular lamellae were seen^32^.

### TUNEL staining

The effect of DEX on TM apoptosis was assessed using the terminal uridyl nick end labeling (TUNEL) in situ cell death detection kit (Roche Diagnostics GmbH, Mannheim, Germany) as per the manufacturer’s instructions. Briefly, the de-paraffinized sections were permeabilized with 0.2% Triton X-100 in 0.1% sodium citrate at 4° C for 2 min and incubated with the provided fluorescein-conjugated TUNEL reaction mixture in a humidified chamber at 37 °C for 1 h in the dark. The TUNEL labeling solution without terminal transferase on tissue section was used as negative control. Tissue sections treated with DNase I served as a positive control. All sections were counterstained with DAPI and examined on fluorescent microscope (AXIO Scope A1, Zeiss, Germany) for the presence of apoptotic positive and non-apoptotic cells in the TM region of the anterior segments.

### Immunofluorescence analysis

Immunohistochemistry was carried out as described previously with some modifications^20^. Briefly, 5 μm tissue sections were de-paraffinized in xylene and rehydrated twice each with 100%, 95% and 75% for 5 min. To unmask the antigen epitopes, heat induced antigen retrieval was performed with 0.1 M citrate buffer, pH 6.4 for 10 min at 95 °C and permeabilized using 0.2% Triton-X100 in PBS for 10 min. Tissue endogenous biotin was blocked using avidin –biotin blocking system for 10 min. Tissue sections were incubated with primary antibody [Myocilin (1:200), R&D Systems (cat# MAB3446), MN, USA; Fibronectin (1:200), Santa Cruz Biotechnology (cat# sc-52331), TX, USA, diluted in 2% BSA] overnight at 4° C in a humidified chamber. Sections were washed thrice with PBS and incubated with secondary antibody [anti mouse IgG (1:200), Santa Cruz Biotechnology (cat# sc-516142), TX, USA] for 4 h. They were then incubated with FITC- labelled streptavidin for 1 h, washed and mounted with anti-fade mounting media containing DAPI (Vector Labs, Inc. CA, USA). Images were captured using fluorescence microscope (AXIO Scope A1, Zeiss, Germany) or confocal microscope (Leica SP8 Confocal Microscope, Leica, Wetzlar, Germany). Tissue sections without primary antibody served as a negative control. Fluorescence images of five consecutive sections of TM from DEX treated and vehicle control eyes were analyzed for myocilin and fibronectin expression (green) in TM. Fluorescence images of TM was carried out for fluorescence intensity quantification using Image J software (National Institute of Health (NIH), Bethesda, USA) with minor modificationsyyy^38^. The fluorescence intensity was background corrected by subtracting with average background intensity values by the formula, as “Corrected Fluorescence Intensity = Fluorescence Intensity in TM—(Area of TM * Mean background fluorescence)/Area of TM”. Five sections per tissue (Responder group, n = 7, Non-Responder group, n = 9 and vehicle treated group, n = 7) were analyzed. The mean fluorescence values of DEX treated group were compared with vehicle treated group.

### Statistical analysis

Statistical analysis was carried out using Graph Pad Prism (ver.8.0.2) (Graph Pad software, CA, USA). All data are presented as mean ± SEM or otherwise specified. Statistical significance between two groups was analyzed using unpaired 2-tailed Student’s *t* test. *p* < 0.05 or less was considered as statistically significant.

## Supplementary Information

Supplementary information.
