# Supplementary material for: Assessment of differential intraocular pressure response to dexamethasone treatment in perfusion cultured Indian cadaveric eyes
Source: Sci Rep. 2021 Jan 12;11:605. doi: 10.1038/s41598-020-80112-8 (PMC7804010; doi:10.1038/s41598-020-80112-8)
Supplement: Supplementary file 1 — Supplementary information. [file 41598_2020_80112_MOESM1_ESM.pdf]

Manuscript Submission Id: 6ecc5a2a-dae5-4d38-b476-9271d1645391

Assessment of Differential Intraocular Pressure Response to Dexamethasone Treatment in Perfusion Cultured Indian Cadaveric Eyes

Ravinarayanan Haribalaganesh[1], Chidambaranathan Gowri Priya[2], Rajendrababu Sharmila[3], Subbaih Krishnadas[3], Veerappan Muthukkaruppan[2], Colin E. Willoughby[4], Srinivasan Senthilkumari[1]*

[1]Department of Ocular Pharmacology, Aravind Medical Research Foundation, #1, Anna Nagar, Madurai-625020

[2] Department of Immunology and Stem cell Biology, Aravind Medical Research Foundation, #1, Anna Nagar, Madurai- 625020

[3] Glaucoma Clinic, Aravind Eye Hospital, #1, Anna Nagar, Madurai-625020

[4] Genomic Medicine Group, Biomedical Sciences Research Institute, Ulster University, Coleraine, Northern Ireland, United Kingdom

* **Corresponding Author:**

Srinivasan Senthilkumari, M. Pharm, Ph. D

Department of Ocular Pharmacology, Aravind Medical Research Foundation

#1, Anna Nagar, Madurai-625020, Tamilnadu, India,

Tele (0): +91-452-4356550; extn. 438; Fax: +91-452-2530984;

e.mail: ss_kumari@aravind.org

Supplementary Table S1: Characteristics of Human Donor Eyes Used for the HOCAS Experiments

DEX Treatment (nM)	Code	Age	Sex	Cause of Death	Time B/W Death & Enucleation (h)	Time B/W Enucleation & Culture (h)	Experiment group	Treatment	Remarks
100	OCHD18-10	76	M	Road traffic injury	1.2	13.6	OD	DEX	Single eye Data included
							OS	-	
	OCHD18-11	79	F	Road traffic injury	0.5	16	OD	DEX	Single eye Data included
							OS	-	
	OCHD18-12	78	F	Natural	3.7	7.3	OD	DEX	Single eye Data included
							OS	-	
	OCHD18-15	55	M	Accident	3.8	23.4	OD	DEX	Single eye Data included
							OS	-	
	OCHD18-16‡	86	M	Respiratory disease	1.5	46	OD	DEX	Data included
							OS	0.1% ETH	
	OCHD18-18	82	M	Respiratory disease	3.5	24	OD	0.1% ETH	Single eye Data included

							OS	-	
	OCHD18-19[‡]	75	M	Cardiac arrest	3	24.5	OD	DEX	Data included
							OS	0.1% ETH	
	OCHD18-20	60	M	Road traffic injury	3	26	OD	-	Single eye Data included
							OS	0.1% ETH	
	OCHD18-24	68	M	Heart disease	4	33.5	OD	-	Single eye Data included
							OS	0.1% ETH	
	OCHD18-27	75	F	Cardiac arrest	1.5	9.5	OD	DEX	Single eye Data included
							OS	-	
	OCHD18-30	75	F	Respiratory arrest	1.5	53.2	OD	DEX	Single eye Data included
							OS	-	
	OCHD18-31	83	M	Heart Disease	1.5	52	OD	DEX	Single eye Data included
							OS	-	
	OCHD18-32	79	M	Respiratory arrest	1.3	51.8	OD	DEX	Single eye Data included
							OS	-	
	OCHD18-34	78	M	Cardiac arrest	2.5	5.5	OD	DEX	Single eye Data included
							OS	-	
	OCHD18-35	80	M	Respiratory arrest	2	52.2	OD	DEX	Single eye Data included

							OS	-	
	OCHD18-36	55	F	Respiratory arrest	1.7	47.4	OD	DEX	Single eye Data included
							OS	-	
	OCHD18-37	53	M	Road traffic injury	4.3	26	OD	DEX	Single eye Data included
							OS	-	
	OCHD18-44	83	F	Cardio Respiratory Arrest	2.5	20.75	OD	DEX	Single eye Data included
							OS	-	
	OCHD19-05‡	78	F	Respiratory disease	3.3	26.1	OD	DEX	Data included
							OS	0.1% ETH	
500	OCHD 17-05	74	M	Respiratory arrest	4	32	OD	-	Single eye Data included
							OS	DEX	
	OCHD 17-06	60	F	Cardiac arrest	3	48	OD	-	Single eye Data included
							OS	DEX	
	OCHD 17-13	83	F	Respiratory arrest	4	63	OD	DEX	Single eye Data included
							OS	-	
	OCHD 18-03‡	51	F	Respiratory arrest	1.5	5	OD	DEX	Data included
							OS	0.1% ETH	
	OCHD 18-05	59	M		2	40	OD	-	Single eye Data included

				Diabetes & Cardiac arrest			OS	0.1% ETH	
	OCHD 18-06	67	F	Cardiac arrest	3	32	OD	-	Single eye Data included
							OS	0.1% ETH	
	OCHD 18-07	77	M	Cardiac arrest	4	25.5	OD	DEX	Single eye Data included
							OS	-	
	OCHD 19-09‡	75	F	Respiratory disease	4.5	20	OD	DEX	Data included
							OS	0.1% ETH	
	OCHD 19-10‡	83	F	Respiratory disease	3.8	30.1	OD	DEX	Data included
							OS	0.1% ETH	
	OCHD 19-14‡	78	F	Natural	1.6	37.6	OD	DEX	Data included
							OS	0.1% ETH	
	OCHD 19-16	80	M	Cardiac arrest	4	7.6	OD	DEX	Single eye Data included
							OS	-	
	OCHD 19-19	69	M	Heart disease	4.2	23.6	OD	DEX	Single eye Data included
							OS	-	
	OCHD 19-20	79	M	Respiratory disease	3.8	27.3	OD	DEX	Single eye Data included
							OS	-	

	OCHD 19-21	73	F	Cerebral vascular accident	5	34.8	OD	DEX	Single eye Data included
							OS	-	
	OCHD 19-23	74	F	Accidental fall	1.5	23.8	OD	DEX	Single eye Data included
							OS	-	
	OCHD 19-24	77	F	Cardiac arrest	3.6	26.5	OD	DEX	Single eye Data included
							OS	-	
	OCHD 19-44	71	M	Respiratory disease	3.5	33.4	OD	DEX	Single eye Data included
							OS	-	

‡ Paired eyes. b/w : between .A total of 43 (7 paired eyes and 29 single eyes) eyes were used for the study with the mean (±) SD age was 73.0 ± 9.50 years. Out of 43 eyes used, 16 eyes received 100nM DEX, 15 eyes received 500nM DEX and 12 eyes received 0.1% ETH as vehicle control. The mean (±) SD elapsed time between (b/w) death and enucleation was 2.86 ± 1.18 h and the mean elapsed time between enucleation and culture was 29.71 ± 14.89 h.

Supplementary Table S2A: The Raw Data of the IOP of Perfusion Cultured Indian Cadaveric Eyes

**Dose group (nM)**	**ETH/R/NR**	**ID**	**Stabilization period (h)**	**Pressure change before and after drug treatment (mmHg)**								**mΔ IOP (mmHg)**
				**D0**	**D1**	**D2**	**D3**	**D4**	**D5**	**D6**	**D7**	
	**ETH**	OCHD18-03	37.8	18.28	21	19.74	20.77	21.36	20.48	18.03	15.59	1.27
		OCHD18-05	33.5	17.16	17.17	17.26	17.59	18.12	19.55	19.11	20.13	1.42
		OCHD18-06	68.3	13.1	15.18	18.74	18.23	17.75	18.29	18.05	19.43	4.85
		OCHD18-16	43.8	17.53	16.32	15.54	17.35	21.78	22.46	18.17	19.06	1.17
		OCHD18-18	41.5	12.04	13.67	13.78	14.21	13.67	16.72	14.96	13.2	2.32
		OCHD18-19	42.1	14	13.21	11.82	9.66	11.18	14.56	17.53	15.79	0.00 [a]
		OCHD18-20	68.6	12.38	12.48	12.14	11.95	12.82	12.87	12.67	11.05	0.00 [a]
		OCHD18-24	75.1	9.31	8.24	7.95	9	8.36	9.04	10.28	11.18	0.00 [a]
		OCHD19-05	41.6	17.42	20.14	25.20	22.43	26.52	19.84	16.36	14.50	3.31
		OCHD19-09	38.3	16.22	16.95	16.51	16.36	15.96	16.45	15.37	14.85	0.00 [a]
		OCHD19-10	43.3	11.19	9.63	10.12	10.08	10.11	10.59	11.45	11.89	0.00 [a]
		OCHD19-14	45.8	10.63	8.88	9.68	8.67	7.75	8.02	9.02	11.38	0.00 [a]
	Baseline IOP (IOP on day 0): mean= 14.1, SD= 3.10, mΔ IOP: mean= 1.19, SD=1.59; N= 12											
**100**	**R**	OCHD18-10	53.3	8.3	12.55	12.61	26.93	28.62	29.27	30.44	31.02	16.19

		OCHD18-11	54.8	14.31	12.44	20.57	44.25	32.22	33.08	34.12	30.16	15.25
		OCHD18-27	61.6	8.97	9.36	22.28	31.51	41.43	45.94	36.98	40.68	23.60
		OCHD18-31	43	25.02	18.9	30.91	45.1	45.89	38.53	33.03	33.15	10.07
		OCHD18-34	45.3	25.42	25.22	23.57	45.86	53.93	52.98	48.47	44.11	16.62
		OCHD18-35	52	15.56	19.01	27.65	26.48	28.86	30.06	28.27	27.65	11.25
	Baseline IOP (IOP on day 0): mean= 16.26, SD= 7.50, mΔ IOP: mean= 15.50, SD=4.79; N= 6											
	**NR**	OCHD18-12	42.6	11.78	7.63	9.24	7.3	9.38	21.21	41.79	-	3.68
		OCHD18-15	40.8	10	10.39	13.61	13.05	10.53	9.4	9.17	9.28	0.78
		OCHD18-16	43.8	15.49	12.05	10.86	11.23	11.52	11.42	11.84	12.33	0.00 [a]
		OCHD18-19	42.1	25.7	26.13	26.82	25.33	24.47	25.22	25.41	25.88	0.00 [a]
		OCHD18-30	43	12.18	11.97	10.59	9.65	13.07	14.38	13.46	14.02	0.35
		OCHD18-32	42.8	20.69	20.23	20.45	23.58	24.17	25.32	24.97	25.6	2.77
		OCHD18-36	33.8	17.09	15.45	13.8	14.6	14.49	16.37	18	20.55	0.00 [a]
		OCHD18-37	69.8	16.1	14.36	12.49	12.56	12.64	12.61	10.98	10.69	0.00 [a]
		OCHD18-44	58.5	23.17	27.07	28.09	28.67	27.7	27.41	26.82	25.95	4.29
		OCHD19-05	41.6	14.98	14.29	13.06	13.36	14.30	14.76	12.38	6.45	0.00 [a]
	Baseline IOP (IOP on day 0): mean= 16.72, SD= 5.09, mΔ IOP: mean= 1.19, SD= 1.71; N= 10											
**500**	**R**	OCHD17-06	36	13.8	12.44	12.37	17.36	24.83	37.76	41.21	-	9.02
		OCHD18-03	37.8	16.01	13.93	28.46	25.65	26.75	27.67	27.12	27.55	9.30

		OCHD18-07	68.3	16.33	23.48	33.26	28.15	36.4	27.81	25.82	18.8	11.67
	Baseline IOP (IOP on day 0): mean= 15.38, SD= 1.38, mΔ IOP: mean= 10, SD= 1.46; N= 3											
	**NR**	OCHD17-05	63.5	14.07	12.01	12.17	14.69	16.85	18.1	18.65	24.83	2.76
		OCHD17-13	40	11.49	11.88	12.5	12.49	14.12	17.57	19.57	22.84	4.35
		OCHD19-09	38.3	24.50	24.25	20.73	20.55	21.24	20.99	20.91	20.38	0.00 [a]
		OCHD19-10	46.3	20.55	20.36	20.85	18.31	16.98	16.68	16.19	17.55	0.00 [a]
		OCHD19-14	45.8	11.82	12.90	12.93	8.24	9.11	9.75	10.37	10.17	0.00 [a]
		OCHD19-16	43.1	18.18	18.78	17.53	16.40	16.81	17.74	19.06	18.88	0.00 [a]
		OCHD19-19	57.5	21.85	18.65	19.10	15.43	21.31	32.77	27.91	28.66	1.41
		OCHD19-20	64.8	19.74	17.93	20.99	18.89	21.88	21.62	20.54	22.12	0.87
		OCHD19-21	40.3	15.91	18.00	17.53	17.03	17.76	17.07	12.72	11.06	0.00 [a]
		OCHD19-23	41.6	13.75	11.21	10.41	10.56	11.96	12.52	12.89	13.74	0.00 [a]
		OCHD19-24	53.8	12.13	11.35	13.93	12.89	11.43	12.73	21.24	24.03	3.37
		OCHD19-44	45.6	17.69	17.10	19.35	21.11	22.73	22.31	21.74	21.46	3.13
	Baseline IOP (IOP on day 0): mean= 16.81, SD= 4.29, mΔ IOP: mean= 1.32±1.64, SD= 2.50; N= 12											

Note: [a] Minus values are given as 0; Day 0 represents the day before respective drug treatments; R - Responder; NR- Non-responder

Supplementary Table S2B: The Raw Data of the Outflow Facility of Perfusion Cultured Indian Cadaveric Eyes

Dose group (nM)	ETH/R/NR	ID	Outflow facility before and after drug treatment (µl/minute/mmHg)								mΔ Outflow facility (µl/minute/mmHg)
			D0	D1	D2	D3	D4	D5	D6	D7	
	ETH	OCHD18-03	0.14	0.12	0.13	0.12	0.12	0.12	0.14	0.16	-0.01
		OCHD18-05	0.15	0.15	0.14	0.14	0.14	0.13	0.13	0.12	-0.01
		OCHD18-06	0.19	0.16	0.13	0.14	0.14	0.14	0.14	0.13	-0.05
		OCHD18-16	0.14	0.15	0.16	0.14	0.11	0.11	0.14	0.13	0.00
		OCHD18-18	0.21	0.18	0.18	0.18	0.18	0.15	0.14	0.19	-0.04
		OCHD18-19	0.18	0.19	0.21	0.26	0.22	0.17	0.14	0.16	0.01
		OCHD18-20	0.20	0.20	0.21	0.21	0.20	0.19	0.20	0.23	0.00
		OCHD18-24	0.27	0.30	0.31	0.28	0.30	0.28	0.24	0.22	0.01
		OCHD19-05	0.14	0.12	0.10	0.11	0.09	0.13	0.15	0.17	-0.01
		OCHD19-09	0.15	0.15	0.15	0.15	0.16	0.15	0.16	0.17	0.01
		OCHD19-10	0.22	0.26	0.25	0.25	0.25	0.24	0.22	0.21	0.02
		OCHD19-14	0.23	0.28	0.26	0.29	0.32	0.31	0.28	0.22	0.05
		Baseline outflow facility (outflow facility on day 0): mean= 0.19, SD= 0.04, mΔ Outflow facility: mean=-0.002, SD=0.03; N= 12									
100	R	OCHD18-10	0.30	0.20	0.20	0.09	0.09	0.09	0.08	0.08	-0.18
		OCHD18-11	0.17	0.20	0.12	0.06	0.08	0.08	0.07	0.08	-0.07

		OCHD18-27	0.28	0.27	0.11	0.08	0.06	0.05	0.07	0.06	-0.18
		OCHD18-31	0.10	0.13	0.08	0.06	0.05	0.06	0.08	0.08	-0.02
		OCHD18-34	0.10	0.10	0.11	0.05	0.05	0.05	0.05	0.06	-0.03
		OCHD18-35	0.16	0.13	0.09	0.09	0.09	0.08	0.09	0.09	-0.07
		Baseline outflow facility (outflow facility on day 0): mean=0.19, SD= 0.09; mΔ Outflow facility: mean= -0.09, SD=0.07; N= 6.									
	**NR**	OCHD18-12	0.21	0.33	0.27	0.34	0.27	0.12	0.06	-	0.02
		OCHD18-15	0.25	0.24	0.18	0.19	0.24	0.27	0.27	0.27	-0.01
		OCHD18-16	0.16	0.21	0.23	0.22	0.22	0.22	0.21	0.20	0.06
		OCHD18-19	0.10	0.10	0.09	0.10	0.10	0.10	0.10	0.10	0.00
		OCHD18-30	0.21	0.21	0.24	0.26	0.19	0.17	0.19	0.18	-0.01
		OCHD18-32	0.12	0.12	0.12	0.11	0.10	0.10	0.10	0.10	-0.01
		OCHD18-36	0.15	0.16	0.18	0.17	0.17	0.15	0.14	0.12	0.01
		OCHD18-37	0.16	0.17	0.20	0.20	0.20	0.20	0.23	0.23	0.04
		OCHD18-44	0.11	0.09	0.09	0.09	0.09	0.09	0.09	0.10	-0.02
		OCHD19-05	0.17	0.17	0.19	0.19	0.17	0.17	0.20	0.39	0.04
		Baseline outflow facility (outflow facility on day 0): mean= 0.16, SD= 0.05; mΔ Outflow facility: mean= 0.01, SD= 0.03; N= 10.									
**500**	**R**	OCHD17-06	0.18	0.20	0.20	0.14	0.10	0.07	0.06	-	-0.04
		OCHD18-03	0.16	0.18	0.09	0.10	0.09	0.09	0.09	0.09	-0.06
		OCHD18-07	0.16	0.11	0.08	0.09	0.07	0.09	0.10	0.13	-0.07

		Baseline outflow facility (outflow facility on day 0): mean= 0.16, SD= 0.02; mΔ Outflow facility: mean= -0.06, SD= 0.01; N= 3.									
	**NR**	OCHD17-05	0.18	0.21	0.20	0.17	0.15	0.14	0.13	0.10	-0.02
		OCHD17-13	0.22	0.21	0.20	0.20	0.18	0.14	0.13	0.11	-0.05
		OCHD19-09	0.10	0.10	0.12	0.12	0.12	0.12	0.12	0.12	0.02
		OCHD19-10	0.12	0.12	0.12	0.14	0.15	0.15	0.15	0.14	0.02
		OCHD19-14	0.21	0.19	0.19	0.30	0.27	0.26	0.24	0.25	0.03
		OCHD19-16	0.14	0.13	0.14	0.15	0.15	0.14	0.13	0.13	0.00
		OCHD19-19	0.11	0.13	0.13	0.16	0.12	0.08	0.09	0.09	0.00
		OCHD19-20	0.13	0.14	0.12	0.13	0.11	0.12	0.12	0.11	-0.01
		OCHD19-21	0.16	0.14	0.14	0.15	0.14	0.15	0.20	0.23	0.00
		OCHD19-23	0.18	0.22	0.24	0.24	0.21	0.20	0.19	0.18	0.03
		OCHD19-24	0.21	0.22	0.18	0.19	0.22	0.20	0.12	0.10	-0.03
		OCHD19-44	0.14	0.15	0.13	0.12	0.11	0.11	0.12	0.12	-0.02
		Baseline outflow facility (outflow facility on day 0): mean= 0.16, SD= 0.04; mΔ Outflow facility: mean= -0.002, SD= 0.03; N= 12.									

Note: D0 represents the day before respective drug treatments; R - Responder; NR- Non-responder
